# Dropout From an Internet-Delivered Cognitive Behavioral Therapy Intervention for Adults With Depression and Anxiety: Qualitative Study

**DOI:** 10.2196/26221

**Published:** 2021-11-12

**Authors:** Kate Lawler, Caroline Earley, Ladislav Timulak, Angel Enrique, Derek Richards

**Affiliations:** 1 E-Mental Health Research Group School of Psychology Trinity College Dublin Dublin Ireland; 2 Clinical Research and Innovation SilverCloud Health Dublin Ireland

**Keywords:** depression, anxiety, iCBT, dropout, internet interventions

## Abstract

**Background:**

Treatment dropout continues to be reported from internet-delivered cognitive behavioral therapy (iCBT) interventions, and lower completion rates are generally associated with lower treatment effect sizes. However, evidence is emerging to suggest that completion of a predefined number of modules is not always necessary for clinical benefit or consideration of the needs of each individual patient.

**Objective:**

The aim of this study is to perform a qualitative analysis of patients’ experiences with an iCBT intervention in a routine care setting to achieve a deeper insight into the phenomenon of dropout.

**Methods:**

A total of 15 purposively sampled participants (female: 8/15, 53%) from a larger parent randomized controlled trial were interviewed via telephone using a semistructured interview schedule that was developed based on the existing literature and research on dropout in iCBT. Data were analyzed using a descriptive-interpretive approach.

**Results:**

The experience of treatment leading to dropout can be understood in terms of 10 domains: relationship to technology, motivation to start, background knowledge and attitudes toward iCBT, perceived change in motivation, usage of the program, changes due to the intervention, engagement with content, experience interacting with the supporter, experience of web-based communication, and termination of the supported period.

**Conclusions:**

Patients who drop out of treatment can be distinguished in terms of their change in motivation: those who felt ready to leave treatment early and those who had negative reasons for dropping out. These 2 groups of participants have different treatment experiences, revealing the potential attributes and nonattributes of dropout. The reported between-group differences should be examined further to consider those attributes that are strongly descriptive of the experience and regarded less important than those that have become loosely affiliated.

## Introduction

### Background

The evidence base supporting internet-delivered cognitive behavioral therapy (iCBT) in the treatment of depression and anxiety is well established [1-5]. However, despite its apparent efficacy, treatment dropout continues to be reported throughout the literature [6]. Treatment dropout is most commonly defined as ending without completing treatment at a predefined cutoff point [7]. Some studies report that just more than half of patients complete a full course of iCBT [8], which is problematic in light of research associating lower completion rates with lower effect sizes [5]. At the same time, we know that it is not necessary for individuals to complete all the per-protocol treatment to benefit clinically [8]. Therefore, what remains is to try to understand dropouts distinctive from the binary classifications of adherence versus nonadherence to a given treatment protocol. Furthermore, considering that the goal of web-based psychological therapies is to provide an evidence-based and cost-effective treatment, help to reduce therapist time and waiting lists, compensate for lack of trained professionals, and alleviate the burden on mental health services of meeting demands [9], it is important to understand web-based treatment dropout to ensure maximal benefit for all involved.

To date, there has been a large body of quantitative research on dropout from web-based psychological therapies that has explored the associated variables to predict which individuals may be more at risk [6,7,10]. Male gender, lower educational levels, self-guided cognitive behavioral therapy (CBT) interventions, and depression with comorbid anxiety symptoms have each been found to significantly increase the risk of dropping out of web-based treatments, whereas the likelihood of dropping out significantly decreases with every additional 4 years of age [6,7]. Our current understanding is also informed by qualitative studies on adherence to internet-delivered psychological therapies [11-13]. These studies have found that the extent to which an individual adheres to a web-based treatment can be largely dependent on their assessment of the advantages and disadvantages of web-based delivery and how this meets their individual needs and preferences [11-13]. Adherers like the freedom and privacy provided by the web-based delivery of psychological interventions, have positive experiences with the content, trust the providers of the web-based program, are motivated to enroll in treatment, consider the web as a substitute for face-to-face support, and feel benefits from the intervention and their use is salient to their need for mental health interventions [11-13]. Their adherence does not appear to be attributed to a singular factor but to a collection of experiences, and their experiences are also not without negatives, citing difficulty with the language of the intervention, lacking confidence in the web-based delivery, a need for face-to-face support, and the potential for difficulties with technology. Although adherence research is a useful starting point for examining dropout, its application is limited insofar, as it cannot be assumed that the opposite will be true for dropout (ie, the disadvantages of web-based interventions explain dropout from them).

More recent research has focused exclusively on qualitatively analyzing individuals’ experiences of web-based treatment dropout [14,15]. Johansson et al [15] reported that web-based treatment dropout is best understood in terms of an incompatible relationship between the perception of treatment and the patient’s situation. A mismatch between any treatment feature and personal prerequisite results in the decision to nonadhere [15], for example, extensive and time-consuming content (treatment features), life factors such as commitments and availability (personal prerequisite), or the lack of face-to-face contact (treatment feature) and the personal preference for a need for face-to-face meetings. Reading and writing demands within the program (treatment feature) and individual capability at these tasks (personal prerequisites) is another example of a potential mismatch. Although a broad picture of web-based treatment dropout begins to emerge across the literature, with each study identifying different influential factors on dropout or suggesting a definitive factor at play, this lack of consensus may allude to the need for a different approach to its exploration.

Efforts to further understand web-based dropout must also take into consideration the conceptualization of the dropout and the implications of this [16]. Högdahl et al [16] summarized that web-based dropout seems to be conceptualized in terms of the number of modules completed rather than the effect of the treatment received on symptoms. This conceptualization may be problematic in light of research stating that treatment completion is not essential for clinical benefit [8]. Furthermore, other researchers argue that dropout is not necessarily a negative outcome or reflective of a wholly negative experience with a web-based intervention [17,18]. They call for patient discretion to be taken into consideration when evaluating and determining dropout status; patients may *drop out* of web-based interventions because they perceive their needs to have been met, and they no longer see the use of staying in treatment regardless of a predefined cutoff point [18,19].

### Objective

The gaps in the web-based treatment dropout literature and the questioning of the current conceptualization suggests that dropout may be more nuanced, with individuals who meet dropout criteria in terms of a predefined number of sessions or modules having widely varied treatment experiences and motives for leaving treatment *prematurely* [17,18]. If this is the case, it is time for the investigation of dropout to move beyond predictors and individual reasons for dropout and look at the whole experience, incorporating the body of existing findings. It is important to ask *dropouts* about their experiences with treatment, exploring all potentially associated factors.

This study aims to conduct an in-depth exploration of the subjective experience of web-based treatment dropout by incorporating current literature on treatment dropout and adherence in both face-to-face and web-based contexts to create a robust semistructured interview. By interviewing and qualitatively analyzing individuals’ experiences of dropout from an iCBT program in a routine care setting, it is hoped that a deeper insight into the experience of treatment dropout will be achieved.

## Methods

### Design

The study was a nested, semistructured qualitative interview study exploring clients’ experiences of dropping out from an iCBT program for depression and anxiety [20]. It was part of a randomized controlled trial (RCT) investigating the effectiveness and cost-effectiveness of internet-delivered interventions for depression and anxiety in the United Kingdom’s Improving Access to Psychological Therapies (IAPT) program [21]. The IAPT program is part of the National Health Service (NHS) designed to provide a stepped care approach for treating people with anxiety and depressive disorders. The results of this RCT showed that the intervention was effective at reducing symptoms of depression and anxiety compared with the waiting-list group, and these effects continued to improve over a 12-month follow-up. In addition, the RCT demonstrated that up to 60% of participants no longer met the criteria for a diagnosis of depression or anxiety at 3 months. With regard to the cost-effectiveness, the intervention was projected to be increasingly cost-effective across the 12-month follow-up horizon [21]. This study followed the COREQ (Consolidated Criteria for Reporting Qualitative Research) guidelines for reporting qualitative research [22]. This study was approved by the NHS England research ethics committee (reference number 17/NW/0311).

### Sample

The larger RCT included 361 individuals; of these 361 individuals, 66.8% (241/361) were randomized to the immediate treatment group and 33.2% (120/361) to the waiting-list control group. The design followed a 2:1 randomization procedure to reduce the likelihood of having many participants waiting for treatment after presenting to the IAPT service. All adult users of the Berkshire NHS Trust IAPT Talking Therapies step-2 services were eligible to participate. Clients were deemed suitable for an internet intervention by their psychological well-being practitioner (PWP) based on their willingness to engage with an iCBT intervention, the presence of mild to moderate levels of anxiety or depression, no suicidal or self-harm risk, and having internet access. In line with the study protocol for the main RCT, a participant was considered to have dropped out of treatment if they received less than 6 web-based reviews from their supporter, as defined by the IAPT.

To identify eligible participants, the lead researcher (KL) manually went through each RCT participant’s iCBT account history from the treatment group to verify the number of modules viewed, reviews received, and how responsive each of these participants was to their research contacts. Their level of responsiveness was determined by their history of answering calls from the RCT research team to complete the research measures. Eligibility criteria included (1) providing written informed consent, (2) completing fewer than 6 reviews with a supporter, and (3) completing a minimum of 1 module. The criterion of completing at least 1 module was necessary so that participants reporting on treatment dropout had some experience with each of the domains of investigation (see *Results*). A Microsoft Excel database was created listing 27 eligible participants for the qualitative interviews. Of the 27 eligible participants, 21 (78%) were invited to participate in the qualitative interviews via telephone at their 6-month or 9-month follow-up for the main RCT before 15 (56%) clients (of the 15 clients, 8/15, 53% women and 7/15, 47% were men) agreed to participate and were recruited. Purposive sampling [23] was used to recruit individuals for the semistructured interviews**.** Following the principles of purposive sampling, it was determined that after the 14th interview, there was a saturation of domains and categories; that is, no new information was being discovered [23]. This was confirmed by the results of the 15th interview. The mean age of participants was 33.5 (SD 9.1) years. The characteristics of the group are summarized in Table 1.

**Table 1:** Characteristics of study participants^a^.

Participant identifier	Gender	Age (years)	Mini-International Neuropsychiatric Interview diagnosis at baseline	iCBT^b^ program	Modules completed, n (%)	Reviews received	Reported reason for change in motivation
P1	Female	24-26	Depression current or past	Space from Depression—8 modules (1 unlockable)	1 (13)	4	Negative reason (not in a receptive frame of mind, contextual obstacles, and iCBT not considered to be personally fitting)
P2	Female	50-53	Depression current or past, panic disorder, and GAD^c^	Space from Depression—8 modules (1 unlockable)	4 (50)	5	Negative reason (not in a receptive frame of mind, contextual obstacles, and iCBT not considered to be personally fitting)
P3	Female	34-36	No diagnosis	Space from Depression and Anxiety—10 modules (2 unlockable)	3 (30)	5	Felt ready to leave treatment early
P4	Female	24-26	Depression current or past	Space from Depression—8 modules (1 unlockable)	7 (88)	3	Felt ready to leave treatment early
P5	Male	30-33	GAD	Space from Depression and Anxiety—10 modules (2 unlockable)	5 (50)	3	Negative reason (iCBT not considered to be personally fitting)
P6	Male	37-39	Depression current or past	Space from Depression and Anxiety—10 modules (2 unlockable)	5 (50)	4	Negative reason (iCBT not considered to be personally fitting)
P7	Male	27-29	Depression current or past and GAD	Space from Depression and Anxiety—10 modules (2 unlockable)	1 (10)	3	Negative reason (not in a receptive frame of mind)
P8	Male	40-43	Depression current	Space from Depression and Anxiety—10 modules (2 unlockable)	7 (70)	2	Did not report
P9	Female	44-46	Panic disorder and GAD	Space from GAD–8 modules (1 unlockable)	4 (50)	2	Negative reason (not in a receptive frame of mind and iCBT not considered to be personally fitting)
P10	Male	44-46	Depression current or past, GAD, and SAD^d^	Space from Depression and Anxiety—10 modules (2 unlockable)	3 (30)	1	Negative reason (iCBT not considered to be personally fitting)
P11	Male	20-23	Depression past	Space from Depression and Anxiety—10 modules (2 unlockable)	4 (40)	4	Felt ready to leave treatment early
P12	Male	20-23	GAD	Space from Depression—8 modules	3 (38)	5	Negative reason (contextual obstacles, and iCBT not considered to be personally fitting)
P13	Female	37-39	No diagnosis	Space from Depression and Anxiety—10 modules (2 unlockable)	1 (10)	4	Did not report
P14	Female	34-36	GAD	Space from GAD—8 modules (1 unlockable)	3 (38)	5	Felt ready to leave treatment early
P15	Female	20-23	Depression current, panic disorder, GAD, and SAD	Space from GAD—8 modules (1 unlockable)	2 (25)	1	Felt ready to leave treatment early

^a^Participants have been allocated participant identifiers P1-P15 to protect their anonymity.

^b^iCBT: internet-delivered cognitive behavioral therapy.

^c^GAD: generalized anxiety disorder.

^d^SAD: social anxiety disorder.

### Treatment

*Space from Depression, Space from Anxiety*, and *Space from Depression and Anxiety* are iCBT interventions for the treatment of depression and anxiety developed by SilverCloud Health with established efficacy [21,24]. These web-based programs comprise 5 core modules: Getting Started introduces CBT and the Thought Feeling Behavior (TFB) cycle, Understanding Feelings focuses on the *feelings* component of the TFB cycle, Spotting Thoughts focuses on the *thoughts* component of the TFB cycle, Challenging Thoughts focuses on taking action against negative and distorted thoughts, and Bringing it All Together prepares the user for coming to the end of the program [24]. *Space from Depression* has 2 additional modules: Boosting Behavior focuses on the inactivity and lack of motivation associated with depression, and Core Beliefs targets the underlying root of unhelpful thoughts that keep the cycle of depression going. *Space from Anxiety* has 2 additional modules: Facing Your Fears focuses on the role of avoidance in maintaining fears and anxiety, and Managing Worry focuses on recognizing real or hypothetical worries and identifying strategies to manage. All modules comprise cognitive and behavioral components, such as self-monitoring, thought recording, behavioral activation, and cognitive restructuring, along with incorporating relaxation exercises and personal stories from past users of the program to help guide clients on how to adapt the learned cognitive and behavioral strategies into their own lives [24].

The programs also use supporters that monitor patients’ progress and provide asynchronous postsession feedback; this is referred to as a *review*. Reviews provide answers to patients’ questions, encouragement and support on work completed and their progress, and signposts them to content. A dashboard interface gives supporters an overview of participants’ level of engagement with the program. Clients in the study from which we recruited our participants were supported by a PWP from the Berkshire NHS Trust IAPT Talking Therapies service. PWPs are graduate psychologists with further training in delivering low-intensity CBT-based interventions [25].

### Measures

#### Mini-International Neuropsychiatric Interview 7.0

The Mini-International Neuropsychiatric Interview 7.0 is a short diagnostic structured interview based on both the Diagnostic and Statistical Manual of Mental Disorders and the International Classification of Diseases criteria. The interview and its administration by telephone have been well validated [26]. For its use during the main RCT, the interview schedule included modules A (major depressive episode), D (panic disorder), F (social anxiety disorder), and N (generalized anxiety disorder) to establish current depression and anxiety and specific anxiety presentations. The Mini-International Neuropsychiatric Interview 7.0 [26] diagnosis was used to assign participants to *Space from Depression, Space from Anxiety,* or *Space from Depression and Anxiety*, with the most suitable intervention being chosen for the participant based on symptomology.

#### Development of the Interview Schedule

**Figure 1:**
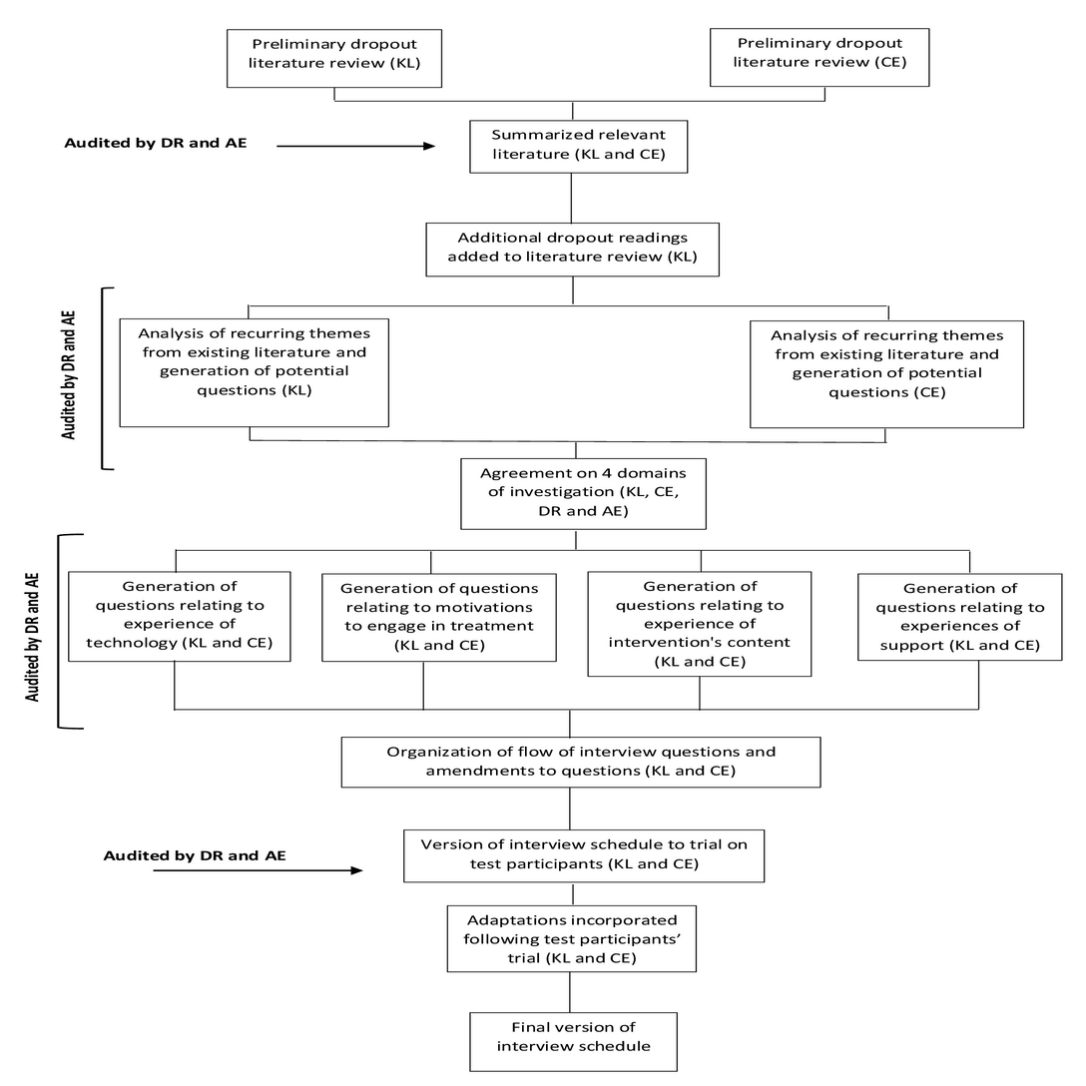
Stages of formation of interview schedule. Author initials are provided parenthetically.

KL and CE reviewed and analyzed the existing literature on treatment dropout to identify the recurring domains of investigation [7,10-15,27]. AE and DR audited this analysis, and 4 broad domains of investigation for treatment dropout were identified: experience of technology, motivations to engage in treatment, experience of intervention’s content, and experiences of support. Questions were generated for each domain of investigation, balancing the greatest number of topics with the least number of questions. The interview was designed in line with the 4 main domains of investigation, and it was concluded that once these 4 domains were interviewed, there would be adequate information to address the research objectives. After discussion, questions were amended and selected for the interview schedule and organized within each domain before trialing the interview with a test participant. The interview schedule was refined once more before the final version was completed (Figure 1).

#### Semistructured Interview

The interview (Multimedia Appendix 1) comprised 22 questions divided into 4 sections: 14% (3/22) questions in the Experience of Technology section (eg, “Did you welcome the intervention being online, considering that you do/don’t use much technology?”), 23% (5/22) questions in the Motivations to Engage in Treatment section (eg, “We note that you completed x sessions and x modules, what changed in this motivation?”), 41% (9/22) questions in Experience of Intervention’s Content section (eg, “Did you feel like the content in the programme was relevant to you?”), and 27% (6/22) questions in the Experiences of Support section (eg, “Everything that you do on the platform you have the option to share with your supporter, how did it feel to communicate in this way?”). This interview schedule provides a ﬂexible framework for the interviews, with scripted prompts for the interviewer. The prompts were included on a side panel of the interview schedule to ensure that the interviewer covered all domains of investigation, checking them off as they went to avoid repetition if a question had already been addressed in a different domain. Prompts also encouraged participants to adequately explore their subjective experiences of treatment dropout and to expand on them if their responses were lacking or they found it difficult to remember.

### Characteristics of Interviewers, Researchers, and Auditors

This research project was led by a researcher with a background in e-Mental Health, who conducted the interviews and analysis (KL). The team of auditors was made up of a researcher undertaking counseling training (CE), a postdoctoral clinical researcher (AE), and a senior researcher (DR) who were all members of the e-Mental Health Research group at the Trinity College Dublin. In addition, a psychologist with a humanistic orientation (LT) who emphasized clients’ agency and interest in psychotherapy research was also a member of the auditor team.

### Procedure

The interviews were conducted by 2 researchers via telephone and lasted between 27 minutes and 67 minutes, depending on the extent to which each participant explored their own experience of treatment dropout. The interviews were recorded and transcribed verbatim by a third-party transcription service. The web-based program was free to access, and participants received a £20 (US $27.32) gift voucher for their participation in the interviews.

### Data Analysis

Data were analyzed using descriptive and interpretive qualitative research methods [28] led by KL. The results were discussed and reﬂected upon with CE, LT, AE, and DR to ensure clarity and consensus on interpretations of the data and their meanings. The method of analysis followed clear steps.

First, the data were divided into discrete meaning units that captured the essence of what participants were trying to convey [29], and irrelevant digressions and repetitions were omitted. Meaning units were coded according to the order in which they occurred and to which participants they belonged. This process provided a clear audit path. All coding of the data was done manually, and Microsoft Excel was used to organize and store the data.

Second, meaning units were assigned to the domains of investigation headings (Experiences of Technology, Motivations to Engage in Treatment, Experiences of Intervention’s Content, and Experiences of Support) to organize the data. The preliminary literature review that informed the creation of the semistructured interview schedule that was used for this study suggested domains of investigation; however, these were not finalized until after the data analysis.

Third, meaning units within the domains were grouped into categories based on their similar meanings. Some meaning units were included in more than 1 category, as they contained more than 1 relevant meaning (therefore, the categories are not mutually exclusive). For example, the meaning unit P2.29 stated the following:

[the supporter] kind of made suggestions...but I didn’t feel [they] was imposing anything on me...[they] emailed something to me that wasn’t on the platform[...] so I really felt they were taking their time to think of what I was going through.

The meaning unit was included in both categories—the category titled *Supporter offered understanding* and the category titled *Supporter tailored treatment to needs*. This process of categorization is subjective and interactive. The data are organized in a way that corresponds with the participants’ meanings while also acknowledging the impact of existing theoretical knowledge [29], as outlined in the background of the interviewers, researchers, and auditors.

Fourth, strategies were used to maintain rigor and credibility. The first author (KL) divided the data into discrete meaning units, and audits were performed at various intervals by the other authors to review this process. The process of organizing meaning units into domains and categories was conducted in several phases. The meaning units were first grouped into domains, and these choices were then discussed with fellow researchers who were experts in the literature, methodology, and iCBT, revising as necessary until agreement was reached. The same method was followed for categorization: the meaning units within each domain were grouped into categories and then presented to fellow researchers for comments and feedback. This process would be repeated until consensus was reached. Records were maintained for each step of the analysis. The feedback provided sometimes outlined a need for clarification of particular meaning units or their reallocation. Using this feedback sometimes resulted in the creation of new domains and categories or the removal of existing domains and categories.

Finally, during data analysis, 2 distinct participant groups emerged, characterized by reasons for the change in motivation to engage with the iCBT treatment: (1) those who felt ready to leave treatment early (5/15, 33%) and (2) those who had negative reasons for their change in motivation (8/15, 53%). Of the 15 participants, 2 (13%) did not report on the reason for their change in motivation to engage with treatment, and so they were excluded from the between-group comparison; however, they contributed to the formation of the overall domains and categories (Table S1 in Multimedia Appendix 2). To compare dropout experiences between the 2 groups, frequency labels were used, as outlined in the consensual qualitative research method [30]. We outline the representativeness of individual categories by considering them *general* if results apply to all cases (ie, 5/5 and 8/8 cases), *typical* if results apply to at least half of the cases (ie, 3-4 of 5 and 5-7 of 8 cases), and *variant* if results apply to fewer than half of the cases (ie, 1-2 of 5 and 1-4 of 8 cases) [30].

## Results

A total of 10 domains capturing the areas of investigation of the subjective experiences of dropout from an iCBT intervention were formulated: relationship to technology, motivation to start, background knowledge and attitudes toward iCBT, change in motivation, use of the program, perceived changes because of the intervention, engagement with content, experience interacting with the supporter, experience of web-based communication, and termination of the supported period (Table S1 in Multimedia Appendix 2). Within each domain, there were positive and negative connotations for the participants’ reports.

### Change in Motivation

Participants who felt ready to leave treatment early (5/15, 33%) reported that they felt they had already obtained what they needed from the treatment without finishing the prescribed number of sessions:

I think it’s just that point I sort of felt like I was getting better. I sort of got what I needed out of [the program]...I was feeling a bit better in my jowls and I didn’t think I really needed it too much.

P15

I got out of it what I needed and...the [supporter] I was speaking to gave me the option just to carry on logging on (my own)...I’m quite comfortable with logging on.

P14

Participants who had negative reasons for their change in motivation (8/15, 53%) responded across 3 categories: not being in a receptive frame of mind, contextual obstacles, and considering iCBT not to be personally fitting:

But also and perhaps because I was just, my brain was just full up of loads of things going on I just wasn't in a receptive frame of mind.

P9

I wasn’t receptive enough to it at the time, but I do think in that frame of mind of feeling so low that you’re kind of not...for months my brain didn’t feel it was working very well.

P2

### Relationship to Technology

Relationship to technology corresponds to technology literacy, familiarity, and usability, both in general and specific to the iCBT program. Participants’ reported relationships with their use of technology in general and with the technology itself were clustered into 10 categories that had both positive and negative connotations (Table 2). For the most part, both groups reported having positive relationships with technology: being familiar with technology, having a sense of anonymity and privacy on the web, and finding the platform easy to use. Negative relationships with technology were considered as a variant or not reported at all: difficulty figuring out how to use the web-based platform, spending too much time on the web, and having poor computer literacy.

**Table 2:** Participants’ relationships to technology based on their reported reasons for their change in motivation.

Domain and categories		Felt ready to leave treatment early (n=5)^a,b^	Negative reason for their change in motivation (n=8)^a,b,c^
**Relationship with technology**			
	Being familiar with technology	General	General
	Sense of privacy and anonymity on the web	General	General
	Good memorability	General	Typical
	Trusted the platform	Typical	Typical
	Easy-to-use web-based platform	Typical	Typical
	Spends too much time on the web	None	Variant
	User dashboard not clear enough	None	Variant
	Layout too structured	None	Variant
	Difficulty figuring out how to use it	Variant	Variant
	Poor computer literacy	Variant	None

^a^Only 13 participants (5/13, 38% felt ready to leave treatment early, and 8/13, 62% had negative reasons for their change in motivation) reported on the reasons for their change in motivation.

^b^General results apply to all cases (ie, 5/5 and 8/8), *typical* results apply to at least half of the cases (ie, 3-4 of 5 and 5-7 of 8), and *variant* results apply to fewer than half of the cases (ie, 1-2 of 5 and 1-4 of 8)

^c^Reported negative reasons for change in motivation to continue engaging with treatment are not being in a receptive frame of mind, contextual obstacles, and internet-delivered cognitive behavioral therapy not considered to be personally fitting.

### Motivation to Start

This category referred to the reasons why participants sought mental health treatment in the first place. All participants reported on their motivations to seek treatment, and their responses were clustered into 2 categories (Table 3)—symptoms of psychological distress and stressful life events:

It was the most severe bout of depression that I’ve experienced. And it scared me, like I felt like I was having thoughts and reacting to things in a way that I couldn’t control.

P4, felt ready

So my husband had just left and I was panicking about like financially I didn’t know what was gonna happen.

P2, negative reason

**Table 3:** Participants’ motivation to start treatment based on their reported reasons for their change in motivation^a^.

Domain and categories		Felt ready to leave treatment early (n=5)^a,b^	Negative reason for their change in motivation (n=8)^a,^^b,^^c^
**Motivation to start**			
	Symptoms of psychological distress	General	General
	Stressful life events	Variant	Typical

^a^General results apply to all cases (ie, 5/5 and 8/8 cases), *typical* results apply to at least half of the cases (ie, 3-4 of 5 and 5-7 of 8 cases), and *variant* results apply to fewer than half of the cases (ie, 1-2 of 5 and 1-4 of 8 cases).

^b^Only 13 participants (5/13, 38% participants felt ready to leave treatment early, and 8/13, 62% participants had negative reasons for their change in motivation) reported on the reasons for their change in motivation.

^c^Reported negative reasons for change in motivation to continue engaging with treatment are not being in a receptive frame of mind, contextual obstacles, and internet-delivered cognitive behavioral therapy not considered personally fitting.

Stressful life events as a motivation to start treatment was a typical category for participants who had negative reasons for their change in motivation but a variant category for participants who felt ready to leave treatment early, indicating that there were some differences between groups with regard to the motivation to start treatment.

### Background Knowledge and Attitudes Toward iCBT

This category was characterized by what participants knew and believed about the iCBT program. Overall, the belief that iCBT could help was typical to both groups:

So when I started the sessions...I thought it would work really well for me because it would be [able to] take my own reflective time and think through my problems.

P5, negative reason

I think I thought that [CBT] could change the way I think.

P12, negative reason

There were between-group differences across the 3 categories (Table 4). A willingness to try the treatment, having no prior knowledge or awareness of CBT, and being skeptical of the treatment approach were typical to participants who felt ready to leave treatment early but variant to those who had negative reasons for their change in motivation:

I didn’t really know what CBT was [...] to a certain extent I didn’t recognize that it would be so much about my thought processes and how it works.

P3, felt ready

**Table 4:** Participants’ background knowledge and attitudes toward iCBTa based on their reported reasons for their change in motivation^b^.

Domain and categories			Felt ready to leave treatment early (n=5)^b,c^		Negative reason for their change in motivation (n=8)^b^^,c,d^
**Background knowledge and attitudes toward iCBT**					
	Belief that iCBT could help	Typical		Typical	
	Willingness to try it	Typical		Variant	
	Had an understanding of CBT^e^	Variant		Variant	
	Trusted provider of web-based treatment	Variant		Variant	
	No prior knowledge or awareness of CBT	Typical		Variant	
	Skeptical of treatment approach	Typical		Variant	

^a^iCBT: internet-delivered cognitive behavioral therapy.

^b^General results apply to all cases (ie, 5/5 and 8/8 cases), *typical* results apply to at least half of the cases (ie, 3-4 of 5 and 5-7 of 8 cases), and *variant* results apply to fewer than half of the cases (ie, 1-2 of 5 and 1-4 of 8 cases).

^c^Only 13 participants (5/13, 38% participants felt ready to leave treatment early, and 8/13, 62% participants had negative reasons for their change in motivation) reported on the reasons for their change in motivation.

^d^Reported negative reasons for change in motivation to continue engaging with treatment are not being in a receptive frame of mind, contextual obstacles, and internet-delivered cognitive behavioral therapy not considered personally fitting

^e^CBT: cognitive behavioral therapy.

### Use of the Program

This category was characterized by reports on how, why, and when participants used the program (Table 5). When use practices were compared between groups, productive and regular use practices were a general category for participants who felt ready to leave treatment early but a variant category for participants who reported negative reasons for their change in motivation:

I set [a reminder] up for like every day at seven o’clock or something...When I’m sitting doing nothing it just gave me a little suggestion to go and do it, I guess.

P11, felt ready

**Table 5:** Participants’ use of the program based on their reported reasons for their change in motivation^a^.

Domain and categories			Felt ready to leave treatment early (n=5)^a,b^		Negative reason for their change in motivation (n=8)^a,b,c^
**Use of the program**					
	Could use it wherever and whenever needed	General		General	
	Productive and regular use	General		Variant	
	Using the program for own benefit	Typical		Variant	
	Could not prioritize time to use it	Typical		Typical	
	Using it out of a sense of obligation rather than for a positive outcome	Variant		Typical	
	Using it when feeling low	Variant		Typical	
	Kept forgetting about the program and appointments	Variant		Variant	

^a^General results apply to all cases (ie, 5/5 and 8/8 cases), *typical* results apply to at least half of the cases (ie, 3-4 of 5 and 5-7 of 8 cases), and *variant* results apply to fewer than half of the cases (ie, 1-2 of 5 and 1-4 of 8 cases).

^b^Only 13 participants (5/13, 38% participants felt ready to leave treatment early, and 8/13, 62% participants had negative reasons for their change in motivation) reported on the reasons for their change in motivation.

^c^Reported negative reasons for change in motivation to continue engaging with treatment are not being in a receptive frame of mind, contextual obstacles, and internet-delivered cognitive behavioral therapy not considered personally fitting.

It was typical for those who had negative reasons for their change in motivation to use it out of obligation or when feeling low:

It felt like obligation. It felt like a tick box exercise.

P6, negative reason

### Perceived Changes Because of the Intervention

Participants’ perceived changes because of the intervention, that is, new skills they acquired and changes to themselves and their everyday lives, were all positive (Table 6). Perceived symptom improvement was viewed as a general category for those who felt ready to leave treatment early but a typical category for those who had negative reasons for their change in motivation:

When my dad did pass away because I was aware of all this stuff that I’ve learned [from the intervention]...And I purposefully the following week, on the exact same day, just to make sure that it [my OCD] wasn’t there, I wore the exact same outfit. To push myself...to prove a point that it’s got nothing to do with what I’m wearing, like it doesn’t matter, it won’t change it.

P3, felt ready

**Table 6:** Participants’ perceived changes because of the intervention based on their reported reasons for their change in motivation^a^.

Domain and categories		Felt ready to leave treatment early (n=5)^a,b^	Negative reason for their change in motivation (n=8)^a,b,c^
**Perceived changes because of the intervention**			
	Symptom improvement	General	Typical
	Applying learned CBT^d^ techniques in everyday life	Typical	Typical
	Developed a knowledge of CBT treatment	Typical	Variant
	Increased awareness or insight	Variant	Variant
	Encouraged to get the help needed	None	Variant

^a^General results apply to all cases (ie, 5/5 and 8/8 cases), *typical* results apply to at least half of the cases (ie, 3-4 of 5 and 5-7 of 8 cases), and *variant* results apply to fewer than half of the cases (ie, 1-2 of 5 and 1-4 of 8 cases).

^b^Only 13 participants (5/13, 38% participants felt ready to leave treatment early, and 8/13, 62% participants had negative reasons for their change in motivation) reported on the reasons for their change in motivation.

^c^Reported negative reasons for change in motivation to continue engaging with treatment are not being in a receptive frame of mind, contextual obstacles, and internet-delivered cognitive behavioral therapy not considered personally fitting.

^d^CBT: cognitive behavioral therapy.

Conversely, being encouraged to get the help they needed was deemed a variant category for those who had negative reasons for their change in motivation, whereas it was not reported by any participants who felt ready to leave treatment early:

I think it was definitely a benefit to kind of like dip my toes in and just get a feel for...cognitive behaviour therapy...it was definitely a good starting point for me.

P12, negative reason

### Engagement With Content

This category was characterized by reports of what participants liked and disliked about aspects of content within the program (Table 7). There were some differences in reporting between the groups. Reflecting back on work completed being beneficial and writing about thoughts and feelings being therapeutic were typical to those who felt ready to leave treatment early:

[I reflected] sometimes, ‘cos if I was having a really bad day and it wasn’t as bad before, it made me feel a little bit better.

P14, felt ready

**Table 7:** Participants’ engagement with content based on their reported reasons for their change in motivation^a^.

Domain and categories		Felt ready to leave treatment early (n=5)^a,b^	Negative reason for their change in motivation (n=8)^a,b,c^
**Engagement with content**			
	Useful tools and exercises	Typical	General
	Reflecting back on completed work was beneficial	Typical	Variant
	Content relevant and relatable to concerns	Typical	Variant
	Manageable workload	Variant	Variant
	Reading and writing provided clarity	Variant	Variant
	Writing about thoughts and feelings felt therapeutic	Typical	Variant
	Felt supported by the program content	Typical	Variant
	Information laid out clearly and concisely	Variant	Variant
	Felt like too much work	Variant	Variant
	Disliked reading and writing	Variant	Variant
	Content was too generic at times	Variant	Variant
	Did not like the personal stories	Variant	Variant
	Content was boring	None	Variant
	Content exacerbated symptoms	None	Variant
	Reflecting of no benefit	None	Variant
	Difficult to understand	None	Variant
	Questionnaires felt pointless	None	Variant
	Did not like the mood monitor	Variant	None
	Content felt disconnected from one section to the next	None	Variant

^a^General results apply to all cases (ie, 5/5 and 8/8 cases), *typical* results apply to at least half of the cases (ie, 3-4 of 5 and 5-7 of 8 cases), and *variant* results apply to fewer than half of the cases (ie, 1-2 of 5 and 1-4 of 8 cases).

^b^Only 13 participants (5/13, 38% participants felt ready to leave treatment early, and 8/13, 62% participants had negative reasons for their change in motivation) reported on the reasons for their change in motivation.

^c^Reported negative reasons for change in motivation to continue engaging with treatment are not being in a receptive frame of mind, contextual obstacles, and internet-delivered cognitive behavioral therapy not considered personally fitting.

However, these categories were deemed variant among those who had negative reasons for their change in motivation*.* Concerning negative experiences engaging with content, reports were low across both groups. Differences emerged with categories such as reflecting being of no benefit, content feeling disconnected between sections and difficult to understand, questionnaires feeling pointless, and finding the content boring or exacerbating to symptoms. There were variant categories among those who had negative reasons for their change in motivation but were not reported at all by those who felt ready to leave treatment early:

[the content] was a bit long winded to be honest with you. There was probably too much reading. So I probably skipped bits.

P10, negative reason

### Experience Interacting With the Supporter

This category relates to participants’ comments on their relationship with their supporters and how they felt that interaction contributed to their overall treatment experience. Participants described these experiences across positive and negative dimensions (Table 8). Feeling supported and connected to their supporter was a general category for those who felt ready to leave treatment early but a variant category for those who had negative reasons for their change in motivation:

I recognise that I’m not looking someone in the face but it turns out to be the same to me because I still felt supported in everything that I did [...] there was just someone there and that to me, was really good.

P3, felt ready

**Table 8:** Participants’ experience interacting with supporters based on their reported reasons for their change in motivation^a^.

Domain and categories		Felt ready to leave treatment early (n=5)^a,b^	Negative reason for their change in motivation (n=8)^a,b,c^
**Experience interacting with supporter**			
	Felt supported by and connected to supporter	General	Variant
	Supporter tailored treatment to needs	Typical	Typical
	Supporter provided a good introduction and explanation of treatment	Variant	Typical
	Felt able to speak freely	Typical	Variant
	Supporter encouraged engagement	Typical	Variant
	Benefitted from having a supporter	Typical	Variant
	Supporter demonstrated a good level of expertise	Typical	Variant
	Supporter discussed treatment goals	Variant	Variant
	Supporter offered understanding	Variant	Variant
	Support felt scripted and impersonal	None	Variant
	Had no sense of connection with supporter	None	Variant
	No feedback from supporter on work completed or messages sent	Variant	Variant
	Supporter never discussed treatment goals and expectations	None	Variant
	Lack of empathy and understanding from supporter	None	Variant
	Lack of guidance from supporter	None	Variant
	Felt like supporter did not care	None	Variant
	Supporter never made contact	None	Variant
	Did not feel comfortable talking with supporter	None	Variant

^a^General results apply to all cases (ie, 5/5 and 8/8 cases), *typical* results apply to at least half of the cases (ie, 3-4/5 and 5-7/8 cases), and *variant* results apply to fewer than half of the cases (ie, 1-2 of 5 and 1-4 of 8 cases).

^b^Only 13 participants (5/13, 38% participants felt ready to leave treatment early, and 8/13, 62% participants had negative reasons for their change in motivation) reported on the reasons for their change in motivation.

^c^Reported negative reasons for change in motivation to continue engaging with treatment are not being in a receptive frame of mind, contextual obstacles, and internet-delivered cognitive behavioral therapy not considered personally fitting.

Furthermore, feeling able to speak freely with their supporter and the supporter demonstrating a good level of expertise was typical to those who felt ready to leave treatment early, whereas these categories were variant to those who had negative reasons for their change in motivation:

They do help you sort of really, really open up and you’ve got to remember, you know, they do this every single day. So, it was quite easy to open up in the first session.

P15, felt ready

Having no connection with the supporter was a variant category for participants with negative reasons for their change in motivation but was not reported by those who felt ready to leave treatment early:

If I had felt a bit more that somebody was really listening and engaging [maybe we could have had a connection]. I just found it hard to build any sort of relationship.

P9, negative reason

Although there was low reporting across the other negative categories, the same pattern applied between groups as with the lack of connection with the supporter.

### Experience of Web-Based Communication

This category was characterized by participants’ likes and dislikes on using a web-based medium to communicate with a supporter. Participants’ reports relating to the medium of web-based communication were described across positive and negative categories, with large differences between groups (Table 9). Liking to communicate on the web with the supporter and finding it easier to open up on the web was typical to those who felt ready to leave treatment early compared with being variant categories for those who had negative reasons for their change in motivation:

I preferred [the online reviews] to be honest. And it was easy enough to do as well.

P14, felt ready

**Table 9:** Participants’ experience of web-based communication based on their reported reasons for their change in motivation^a^.

Domain and categories			Felt ready to leave treatment early (n=5)^a,b^		Negative reason for their change in motivation (n=8)^a,b,c^
**Experience of web-based communication**					
	Frequency of web-based communication worked well	Typical		Typical	
	Liked communicating web-based with supporter	Typical		Variant	
	Easier to open up on the web and feeling of disinhibition	Typical		Variant	
	Preference for face-to-face communication	None		Typical	
	Needed more contact with supporter	Variant		Variant	
	Communicating on the web was too formal and structured	None		Typical	
	Lack of instantaneous responding with supporter	None		Variant	
	Could not open up to a computer	None		Variant	
	Web-based communication felt too anonymous	None		Variant	

^a^General results apply to all cases (ie, 5/5 and 8/8 cases), *typical* results apply to at least half of the cases (ie, 3-4 of 5 and 5-7 of 8 cases), and *variant* results apply to fewer than half of the cases (ie, 1-2 of 5 and 1-4 of 8 cases).

^b^Only 13 participants (5/13, 38% participants felt ready to leave treatment early, and 8/13, 62% participants had negative reasons for their change in motivation) reported on the reasons for their change in motivation.

^c^Reported negative reasons for change in motivation to continue engaging with treatment are not being in a receptive frame of mind, contextual obstacles, and internet-delivered cognitive behavioral therapy not considered personally fitting.

Conversely, a preference for face-to-face interactions was a typical category for those who had negative reasons for their change in motivation but not reported at all by those who felt ready to leave treatment early:

I think looking back that maybe I should’ve had both, even though I was short on time, actually the (face-to-face) probably would’ve been better than maybe moving to [iCBT.].

P1, negative reason

### Termination of Supported Period

This category was characterized by participants’ reports relating to how the supported period of the iCBT program was discontinued and how they felt about it (Table 10). It was typical for participants from both groups to feel able to go back to this treatment if they felt the need in the future:

If I explored a different route and it didn't work out then I was always welcome to rejoin SilverCloud, or rejoin Talking Therapies...So that is really positive.

P5, negative reason

Being happy with how the supported period ended was a general category for those who felt ready to leave treatment early but was not reported at all by those who had negative reasons for their change in motivation:

I think I got out of it what I needed...[my supporter] gave me the option just to carry on logging on (without support) and I’m quite comfortable with logging on.

P14, felt ready

More negative reports relating to the termination of the supported period were variant categories for those who had negative reasons for their change in motivation, whereas they were not reported by those who felt ready to leave treatment early: support stopping unexpectedly and feeling abandoned and feeling relieved that support stopped because of it being such a negative experience:

[Support] stopped. I heard nothing, done nothing...I was shocked and disappointed.

P10, negative reason

I haven't got time for this, you're not useful enough to me. Therefore, I'm not wanting to carry it on and give you my time because my time was too precious and as I say it just wasn't useful enough.

P9, negative reason

**Table 10:** Participants’ experience of termination of the supported period based on their reported reasons for their change in motivation^a^.

Domain and categories			Felt ready to leave treatment early (n=5)^a,b^		Negative reason for their change in motivation (n=8)^a,b,c^
**Termination of supported period**					
	Feels able to go back to treatment if needed	Typical		Typical	
	Happy with how support was terminated	General		None	
	Had a conversation with supporter about finishing treatment	Variant		Variant	
	No longer a priority, just let it go	Variant		Variant	
	Support stopped unexpectedly, felt abandoned	None		Variant	
	Felt relieved that support stopped as it was a negative experience	None		Variant	

^a^General results apply to all cases (ie, 5/5 and 8/8 cases), *typical* results apply to at least half of the cases (ie, 3-4 of 5 and 5-7 of 8 cases), and *variant* results apply to fewer than half of the cases (ie, 1-2 of 5 and 1-4 of 8 cases).

^b^Only 13 participants (5/13, 38% participants felt ready to leave treatment early, and 8/13, 62% participants had negative reasons for their change in motivation) reported on the reasons for their change in motivation.

^c^Reported negative reasons for change in motivation to continue engaging with treatment are not being in a receptive frame of mind, contextual obstacles, and internet-delivered cognitive behavioral therapy not considered personally fitting.

## Discussion

### Principal Findings

This study qualitatively investigated dropout from iCBT interventions for depression and anxiety as part of routine mental health service delivery. It explored dropout across a continuum of 10 experiential domains. These domains are multiple and varied and demonstrate the conceptualization of treatment dropout as an experience not confined to one moment. Furthermore, this study establishes that when we conceptualize dropout in terms of the number of sessions completed, there are 2 distinct groups of participants: those with negative reasons for their change in motivation and those who feel ready to leave treatment early. However, the differences in treatment experiences observed between these groups point to a potential shift in how we think about treatment dropout.

This study has taken a deeper dive into dropout from iCBT treatment research. Previously, personal characteristics, individual capabilities, aspects of technology, intervention content, relationship with the supporter, motivation and treatment expectancies, and credibility have all been identified as reasons for treatment dropout [6,7,14,15]. This study examined dropout across the complete treatment experience and now recognizes it as a concept with related attributes and nonattributes, that is, treatment factors and experiences that describe it. The findings presented, although preliminary and requiring validation on a larger scale, advance what we know of dropout by suggesting that certain attributes of the treatment experience are strongly descriptive of dropout, whereas others may need to be reconsidered. To be descriptive of dropout, an attribute serves to describe the experiences of iCBT treatment leading up to dropout that were instrumental to the reason for dropout; therefore, a nonattribute is not pertinent to the decision to drop out.

Considering that participants described both their relationship with technology in general and to the iCBT program specifically as largely positive and that there were little to no differences in reporting between the 2 groups, it should be further considered whether technology is now a nonattribute of treatment dropout. To date, the literature has reported it to play an important role in dropout in terms of technology literacy, attitudes toward the technologization of health care, and technical difficulties [7,11-14,27]. Perhaps this is because after decades of development, design and technical flaws have been rectified [31,32], and technology and the internet have become both pervasive and accessible. These findings were echoed in the background knowledge and attitudes toward the iCBT programs domain, rendering it a nonattribute of treatment dropout. Individuals were mostly accepting of the use of technology in the delivery of their mental health care. Perhaps the routine care setting where iCBT is offered as a reliable treatment alternative may have acted as a buffer against the nonacceptance of and negative attitudes toward internet interventions [33-35].

Stressful life events before beginning iCBT treatment seem to be an attribute of dropout because proportionately more participants who had negative reasons for their change in motivation reported them than those who felt ready to leave treatment early. This is not surprising, as one of the main characteristics of this group is not continuing with treatment because of contextual obstacles such as work, relationships, and commitments. The literature has previously documented the influence of external factors on treatment dropout [8,10,15,36], stating that the demands they place on the individual will lead to dropout if viewed as an obstacle to their daily life [15]. Although these participants reported these life events as triggers for seeking and beginning treatment, they may have contributed to suboptimum conditions to continue treatment. The descriptive characteristics of stressful life events for dropouts have implications for the clinical application of iCBT treatment. They may indicate a need for greater consideration to be given to suitability screening or increased pretherapy effort and tailoring (including content type and duration of treatment) of treatment to counteract them.

On the basis of the findings reported in relation to *perceived changes because of the intervention,* all participants perceived some benefit from the iCBT program, with the most commonly reported change being perceived symptom improvement, an attribute consistent with the delivery of an effective treatment in any format. This preliminary finding may align with the Waller and Gilbody [8] conclusion that treatment completion is not always necessary for clinical benefit and gives support to the Eysenbach [18] and Proudfoot [37] hypothesis that dropout is not necessarily reflective of a wholly negative experience. Furthermore, they suggest that perceived changes because of the intervention are attributes of treatment dropout. Although there may be reason to question the exclusivity of positive outcomes to treatment completion, these findings are tentative and largely based on patient discretion, and therefore, further research would be required to reach a conclusive decision regarding the relationship between dropout and positive outcomes. Interestingly, proportionately more participants with negative reasons for their change in motivation reported that the iCBT treatment encouraged them to receive the help they needed than those who felt ready to leave treatment early. This was considered a positive outcome as effective treatments work to leverage aspects of treatment, which prompts further help-seeking behaviors [38,39]. Further studies are required to explore this relationship between dropout and positive outcomes.

Negative interactions with a supporter and a lack of a connection characterize the dropout experience of those who had negative reasons for their change in motivation and who, according to the consensual qualitative research method categorizations [40], reported more than their counterparts across these categories. The differences observed between the 2 groups in terms of their experiences with supporters validate the emphasis placed on the patient-clinician relationship for treatment success [41,42] in the existing literature [7,10,11,13-15,27]. Although it is evident from previous research and the reports of those who felt ready to leave treatment early that this alliance can be established on the web [41,43], it may have been the case for participants who had negative reasons for their change in motivation that they could not overcome the altered dynamics of moving the relationship on the web [17,44]. From the findings of this study, it is clear that a poor-quality clinician-patient relationship facilitates dropout, rendering it an attribute.

There were also differences in reporting between groups in relation to the experience of web-based communication. A dislike for web-based communication and a preference for face-to-face treatment characterizes the dropout experiences of those who had negative reasons for their change in motivation. This finding is reflective of the pattern in reporting experiences with the supporter, and it would be interesting to investigate whether they are correlated. The role played by preferences in treatment dropout has been identified previously, concluding that despite the comparable efficacy of web-based communication with a supporter, an overwhelming number of patients only prefer face-to-face interaction [43,45-47]. Considering the presence of such preferences in this study and previous research stating that if the support or communication type is not compatible with the patient’s preferences or expectations, the patient may decide to drop out of treatment [15,48], a dislike for web-based communication and a preference for face-to-face treatment is a strongly descriptive of dropout, positing it as an attribute of treatment dropout. Further research should explore whether this dislike for web-based communication is a true dislike or whether it speaks more to the fact that, for these individuals, web-based communication is just not enough and should be provided in combination with face-to-face interaction.

The multiplicity and variance of the domains presented in this study expand our understanding of dropout. This nuanced portrayal was achieved through a robust methodology consisting of the development of a semistructured interview based on the existing literature pertaining to dropout and adherence in both face-to-face and web-based therapies and rigorous analysis using the descriptive-interpretive method [28]. It demonstrates the complexity of the dropout experience, calling into question the validity of conceptualizing treatment dropout in terms of compliance to modules or sessions needing completion, as some research does [16,49]. This has implications for both iCBT and clinical practice. As presented in the comparison between the 2 groups, some aspects of the treatment experience have become highly relevant to dropout: stressful life events before beginning treatment, using the iCBT program when feeling low or out of a sense of obligation, perceived changes because of the intervention, negative experiences with content, negative experiences with the supporter, a dislike for web-based communication, and a preference for face-to-face therapy, whereas others have diminished into the background or disappeared entirely: relationship to technology, background knowledge and attitudes toward iCBT, and termination of the supported period. It is apparent that all participants who dropped out benefitted somewhat; what needs to be understood now is who benefits the most, and these findings can help guide this future research. Furthermore, as the future of digital health care depends on the increased understanding of such phenomena so that psychological interventions can continue to increase in accessibility while increasing specificity for the patient, research exploring the complete experience is necessary.

### Limitations

Although the ecological validity provided by the IAPT setting was a strength of this study, it may also have positively skewed participant reports, as their suitability for iCBT would have been assessed before beginning treatment. In addition, some individuals did not want to participate in the dropout interview. By not capturing the experiences of these individuals, the data presented may be positively biased to the intervention. The between-group differences that have been identified are based on qualitative data from a sample of 15 participants who dropped out in response to open-ended questions rather than closed-ended questions that would have investigated the presence or absence of an experience and so should be considered tentative. Future research into dropout should focus on identifying these 2 groups of participants on a larger scale and quantitatively investigate their outcomes. As with any qualitative interview study, the potential roles played by social desirability, historical reporting, and researcher subjectivity should be taken into account. However, the results for all 15 participants were analyzed first, and it was determined only afterward that a second analysis with the participants divided into 2 groups would be useful to avoid any potential bias from the researcher.

### Conclusions

The data presented from the qualitative interviews provide insight into the subjective experiences of participants who dropped out from an iCBT treatment for depression and anxiety in a routine care setting. In doing so, it moved beyond the current understanding of treatment dropout as a seemingly negative outcome attributable to a singular event and presents it as a phenomenon that must be considered experientially. The findings bring to light a more nuanced picture of treatment dropout when looked at through the perspective of varied domains that shed light on the experience. This suggests that participants who drop out can be distinguished in terms of their change in motivation: those who felt ready to leave treatment early and those who had negative reasons for dropping out. In doing so, it facilitated a comparison of treatment experiences that revealed potential attributes (stressful life events before beginning treatment, using the iCBT program when feeling low or out of a sense of obligation, perceived changes because of the intervention, negative experiences with content, negative experiences with the supporter, a dislike for web-based communication, and a preference for face-to-face therapy) and nonattributes (relationship to technology, background knowledge and attitudes toward iCBT, and termination of the supported period) of dropout. To understand why individuals drop out, these between-group differences should be examined to consider those features that are strongly descriptive of the experience and regard those that have become loosely affiliated with less importance. The evidence presented in this study stipulates that there is a difference between what we label as a dropout and what should actually be considered a dropout. Further work, either quantitative or exploratory, is needed to comprehensively develop a typology of dropout participants and potentially reconceptualize the phenomenon in this rapidly changing digital health care setting.

## Appendix

Multimedia Appendix 1 Semistructured interview schedule exploring the experience of dropout from an internet-delivered cognitive behavioral therapy intervention.

Multimedia Appendix 2 Participants’ experiences of treatment based on their reported reasons for their change in motivation.

## Abbreviations

CBT: cognitive behavioral therapy
COREQ: Consolidated Criteria for Reporting Qualitative Research
IAPT: Improving Access to Psychological Therapies
iCBT: internet-delivered cognitive behavioral therapy
NHS: National Health Service
PWP: psychological well-being practitioner
RCT: randomized controlled trial
TFB: thought feeling behavior
