# Supplementary material for: Dropout From an Internet-Delivered Cognitive Behavioral Therapy Intervention for Adults With Depression and Anxiety: Qualitative Study
Source: JMIR Form Res. 2021 Nov 12;5(11):e26221. doi: 10.2196/26221 (PMC8663602; doi:10.2196/26221)
Supplement: Multimedia Appendix 1 [file formative_v5i11e26221_app1.pdf]

Interview layout

1. Intro
2. Inform of interview duration, purpose and format.
3. Ask prepared questions - probe
4. Closing questions; risk; trial; summarise main issues – agree, add/subtract
5. Explain next steps; Payment & next batch of main trial questionnaires
6. Thank you.
7. Send debriefing sheet & payment

Phone script

Hello, can I speak to __________ please?

Hi, my name is ____________ and I'm calling from the SilverCloud research team. Before I continue would you please confirm your date of birth?

And your address is still __________? We just want to make sure that your payment for this interview is sent to the correct address.

Before I go on to the interview itself, I just want to make you aware that while calls are confidential, there are exceptions to this, such as if I were to feel that you or someone else were at risk in any way then I may have to tell someone to make sure whoever is at risk is safe.

This interview will be exploring your experience of an online intervention. It will take about 30 minutes to an hour and the open-ended questions will provide you with plenty of opportunity to give feedback. For the questions we would ask that you don't think too long on your answers but go with what comes to mind first. There are no right or wrong answers and we would ask that you answer as honestly as possible.

I may interrupt you from time to time to ensure we cover all the questions today and keep to time. Is that okay with you?

The interview is divided into 4 sections and I will let you know as we progress from one stage to the next. The interview is being recorded for transcription purposes and will be held confidentially as outlined in the information sheet you received previously.

This first section is based on technological aspects of the intervention. Let's begin…

The first section is based on the online platform. Let's begin…

Computer literacy ☐

T1. Do you make use of technology much in your daily life? ☐

Fatigue ☐
Attitudes ☐
Perceived credibility ☐
Familiarity with content ☐
Beliefs/perceptions ☐
Past experience ☐
Preferences ☐
Scepticism ☐

T2. Did you welcome the intervention being online, considering that you do/don't use much technology? ☐

Memorability/picking back up ☐
Learnability ☐
Error recovery ☐
Trust in the brand ☐

How did you find navigating around the platform? ☐

Frustration/irritability/tiredness ☐
Concentration ☐
Side effects from platform ☐

Tech or symptom related difficulties? ☐

Privacy on the platform ☐
Stigma ☐
Anxiety around privacy issues ☐
(Paranoia – where info. going) ☐
Trust in the brand ☐
Perceived anonymity/distance ☐
Disinhibition effect ☐

T3. So, we talked a little there about the technical aspects of the platform. With the intervention being online, did you find it to be more private or was there privacy issues that were concerning to you? ☐

Privacy in usage ☐
Access ☐

Could you always access a computer privately? ☐

Social support ☐

Did you feel supported in your decision to engage in the programme? ☐

Motivation ☐<br>Personal factors ☐<br>Psychological mindedness ☐	*So, now we are going to just talk a little about your own use of the programme…*<br><br>M1. Can you tell me a little bit about what motivated you to use the intervention in the first place? ☐
Depth of learning in modules done ☐<br>Why was what done, enough ☐<br>Any different if F2F ☐<br>Effort ☐<br>Priorities ☐<br>Needed low mood/distress ☐<br>Exacerbated symptoms ☐<br>Side effects ☐<br>Preferences ☐	[If unmentioned] We note that you completed x sessions and x modules, what changed in this motivation? ☐
Not needed/improved – how/why ☐<br>Not needed/not improved – how/why ☐<br>Exacerbated symptoms ☐<br>Side effects ☐	M2. So, you were committed to using the programme? OR you were more committed to X and Y? ☐<br><br>You made it fit into your day? ☐
Weekly/sparse ☐<br>Forgetting/reminders ☐<br>Using when low/distress ☐<br>Commitment/discipline ☐	M3. Do you think you were improving by using the intervention or not improving? ☐<br><br>M4. When did you typically use SilverCloud? ☐
Commitment ☐<br>Priorities ☐<br>Procrastination ☐<br>Side effects e.g. overwhelm ☐<br>Access/logistics ☐<br>Preferences ☐	How did you organise your using it? ☐<br><br>Was it for yourself or did you feel like you had to? (Example F2F CBT)

Usage since dropout □<br>Why □<br>What □<br>How □	M5. Have you continued to make use of the techniques or content since stopping treatment? □
	Now, we are going to talk a little about you, your symptoms and the content in the programme…
Locus of control/sense of responsibility □<br>Symptom length/severity □<br>Perceived credibility □	C1. BEFORE you began the intervention did you feel that your symptoms were manageable at all? □
	C2. When you STARTED the intervention did you feel that you would be able to manage your symptoms better by using the programme? □
Psychological mindedness □<br>Prior experience □<br>Stigma □<br>Perceived credibility □	C3. As you know, the programme is based on Cognitive Behavioural Therapy, what was your understanding of CBT at the time? □
Identification with CBT □<br>Agreed upon goals □<br>Support □<br>Familiarity □	Did you feel that CBT could help you? □
Prior experience with an intervention □	C4. Had you engaged in any other psychological treatments before SilverCloud? □

Preference □<br>Perceived credibility □<br>Stigma □<br>II or therapy in general □<br>Preference for f2f why □<br>What expect from F2F □	C5. Would you have any preference on treatment approach? □
Relevance □<br>Symptom length/severity □<br>Comorbidity □<br>Familiarity with content □<br>Preference □	C6. And did you feel like the content in the programme was relevant to you? Why? □<br><br>How did it feel to work through? □
Tailoring □<br>Expectations □<br>Side effects e.g. discomfort with issues raised/overwhelm □<br>Psychological mindedness □<br>Rationale for treatment □	So, you felt/didn't feel like a lot of it addressed ***your*** concerns? Could you tell me a little bit more about that? □
Mind-set changed □<br>Difficulty applying strategies, why □<br>Tailoring □<br>Alternative better □<br>Using when low/distressed □	C7. Did you feel like you could apply or follow the strategies in your daily life? □
Locus of control/sense of responsibility □<br>Content □<br>Support □<br>Alternatives better, how □	C8. Throughout the programme, did you feel that it met your expectations? □<br><br>With regards the level of work you had to put in? □

Mechanisms ☐
Operational ☐
Preferences ☐
Reading/writing capabilities ☐
Expectations around F2F CBT ☐
Side effects ☐
Psychological mindedness ☐

Anonymity/distance ☐

Privacy ☐

Connectedness ☐

Responsiveness ☐

Alliance ☐

Support quality ☐

Responsiveness ☐

Supporter expertise ☐

Tailoring ☐

Preference ☐

Cancellations/rupture ☐

Could tell supporter ☐

Rigid/flexible ☐

Common understanding of aims ☐

Treatment rationale ☐

Therapist expertise ☐

Alliance/sense of connectedness ☐

Tailoring ☐

Sense of obligation/for self ☐

C9. So, we have talked about the content, how did it feel to read content and type or write about your thoughts, feelings and behaviours? ☐

Do much of it? ☐

More opportunity for reflection? ☐

Look back on/future use? ☐

Pros & Cons? ☐

S1. As you know, everything that you do on the platform you have the option to share with your supporter, I wonder how did it feel to communicate in this way? ☐

S2. How did you find the contacts/reviews? ☐

What would you have liked it to be like? Or how could it have been better? ☐

How did you find the frequency of the reviews? Did this work with your schedule? ☐

You felt like your supporter had the same goals in mind for you/had different ideas as to why you signed up? ☐

Did you feel connected to him/her? ☐

Encouraged adherence □

Applicability to life □

Discipline/commitment □

Psychological mindedness □

Alliance □

S3. [In light of the above – reflect] – did this affect your use of the platform and content? □

The end of the supported period….

Alliance □

Access □

Felt let down □

Felt ready to go/empowered □

How was that reached? □

How did **you feel** about that? Did you get what you needed? □

Did you feel like you would be able to ask for something different if you wanted to? □

Disappointing – Self/service/self □

How □

Why □

Feel able to go back for further support if you need to? □

Were you able to ask for what you needed? □
(remember distinction between disappointment between service/SCH/self

What would have helped? □

Expectations □

Perceptions □

Introduction to treatment □

Willingness to engage □

Attitude □

S5. So, in light of how it went and ended, was this in line with how the programme was introduced at the beginning and were your expectations met? □

Delay in TS1 □<br>Introduction to programme □<br>Perceptions □<br>Perceived credibility □	S6. Did you start treatment immediately? □<br><br>How did this affect your use of the platform? □
Introduction □<br>Therapist expertise □	Was the first session helpful in getting you started? Did you feel confident and hopeful in your supporter? □
Agreed rationale □<br>Comorbidity □<br>Symptom severity/length □<br>Introduction to programme □<br>Expectations □	Did you feel that the approach was right for you at the time? □
Barriers □<br>Stigma □<br>Mind-set changed □	Did you find that this treatment removed barriers to treatment that you would have met otherwise? □
Risk	I can see on your __________ follow-up point, that you mentioned thoughts of ending your life or of harming yourself in some way. This can be a very normal response to feeling down or anxious. Did you find the supports around this sufficient or was the programme helpful for you in this in any way? □
Trial	Did participating in the research trial affect your engagement with the treatment? □

Summary

We have come to the end of the interview. To summarise, you have mentioned (See 4 sections above).

Would you agree with all of this? ☐

Is there anything you would like to add/subtract? ☐

What would have helped? ☐

Do you have any questions for me before we go?

Thanks again for taking the time to do this interview. As a token of our appreciation we will be sending you a One4All voucher, which you should receive shortly.

Your participation in the main trial will soon be over also. You have ____________ batches of questionnaires yet to complete. The next ones will be available to you around ___________ and you can complete these online. This will add value to another One4All voucher. Thanks again. Bye.
