# Supplementary material for: Dropout From an Internet-Delivered Cognitive Behavioral Therapy Intervention for Adults With Depression and Anxiety: Qualitative Study
Source: JMIR Form Res. 2021 Nov 12;5(11):e26221. doi: 10.2196/26221 (PMC8663602; doi:10.2196/26221)
Supplement: Multimedia Appendix 2 [file formative_v5i11e26221_app2.docx]

Table 11. Participants’ experiences of treatment based on their reported reasons for their change in motivation^a-c^.

|  |  | Change in motivation (n=13) | |
| --- | --- | --- | --- |
| Domain | Categories | Felt ready to leave treatment early  (n=5) | Negative reason for their change in motivation (n=8) |
|  |  |  |  |
| Relationship with technology |  |  |  |
|  | Being familiar with technology | General | General |
|  | Sense of privacy and anonymity online | General | General |
|  | Good memorability | General | Typical |
|  | Trusted the platform | Typical | Typical |
|  | Easy-to-use online platform | Typical | Typical |
|  | Spends too much time online | None | Variant |
|  | User dashboard not clear enough | None | Variant |
|  | Layout too structured | None | Variant |
|  | Difficulty figuring out how to use it | Variant | Variant |
|  | Poor computer literacy | Variant | None |
| Motivation to start |  |  |  |
|  | Symptoms of psychological distress | General | General |
|  | Stressful life events | Variant | Typical |
| Background knowledge and attitudes towards iCBT |  |  |  |
|  | Belief that iCBT could help | Typical | Typical |
|  | Willingness to try it | Typical | Variant |
|  | Had an understanding of CBT | Variant | Variant |
|  | Trusted provider of online treatment | Variant | Variant |
|  | No prior knowledge or awareness of CBT | Typical | Variant |
|  | Sceptical of treatment approach | Typical | Variant |
| Usage of the programme |  |  |  |
|  | Could use it wherever and whenever needed | General | General |
|  | Productive and regular use | General | Variant |
|  | Using the programme for own benefit | Typical | Variant |
|  | Couldn’t prioritise time to use it | Typical | Typical |
|  | Using it out of a sense of obligation rather than for a positive outcome | Variant | Typical |
|  | Using it when feeling low | Variant | Typical |
|  | Kept forgetting about the programme and appointments | Variant | Variant |
| Perceived Changes due to the intervention |  |  |  |
|  | Symptom improvement | General | Typical |
|  | Applying learned CBT techniques in everyday life | Typical | Typical |
|  | Developed a knowledge of CBT treatment | Typical | Variant |
|  | Increased awareness and/or insight | Variant | Variant |
|  | Encouraged to get the help needed | None | Variant |
| Engagement with content |  |  |  |
|  | Useful tools and exercises | Typical | General |
|  | Reflecting back on completed work was beneficial | Typical | Variant |
|  | Content relevant and relatable to concerns | Typical | Variant |
|  | Manageable workload | Variant | Variant |
|  | Reading and writing provided clarity | Variant | Variant |
|  | Writing about thoughts and feelings felt therapeutic | Typical | Variant |
|  | Felt supported by the programme content | Typical | Variant |
|  | Information laid out clearly and concisely | Variant | Variant |
|  | Felt like too much work | Variant | Variant |
|  | Disliked reading and writing | Variant | Variant |
|  | Content was too generic at times | Variant | Variant |
|  | Didn’t like the personal stories | Variant | Variant |
|  | Content was boring | None | Variant |
|  | Content exacerbated symptoms | None | Variant |
|  | Reflecting of no benefit | None | Variant |
|  | Difficult to understand | None | Variant |
|  | Questionnaires felt pointless | None | Variant |
|  | Didn’t like the mood monitor | Variant | None |
|  | Content felt disconnected from one section to the next | None | Variant |
| Experience interacting with supporter |  |  |  |
|  | Felt supported by and connected to supporter | General | Variant |
|  | Supporter tailored treatment to needs | Typical | Typical |
|  | Supporter provided a good introduction and explanation of treatment | Variant | Typical |
|  | Felt able to speak freely | Typical | Variant |
|  | Supporter encouraged engagement | Typical | Variant |
|  | Benefitted from having a supporter | Typical | Variant |
|  | Supporter demonstrated a good level of expertise | Typical | Variant |
|  | Supporter discussed treatment goals | Variant | Variant |
|  | Supporter offered understanding | Variant | Variant |
|  | Support felt scripted and impersonal | None | Variant |
|  | Had no sense of connection with supporter | None | Variant |
|  | No feedback from supporter on work completed or messages sent | Variant | Variant |
|  | Supporter never discussed treatment goals and expectations | None | Variant |
|  | Lack of empathy and understanding from supporter | None | Variant |
|  | Lack of guidance from supporter | None | Variant |
|  | Felt like supporter didn’t care | None | Variant |
|  | Supporter never made contact | None | Variant |
|  | Didn’t feel comfortable talking with supporter | None | Variant |
| Experience of online communication |  |  |  |
|  | Frequency of online communication worked well | Typical | Typical |
|  | Liked communicating online with supporter | Typical | Variant |
|  | Easier to open up online, feeling of disinhibition | Typical | Variant |
|  | Preference for face-to-face communication | None | Typical |
|  | Needed more contact with supporter | Variant | Variant |
|  | Communicating online was too formal and structured | None | Typical |
|  | Lack of instantaneous responding with supporter | None | Variant |
|  | Couldn’t open up to a computer | None | Variant |
|  | Online communication felt too anonymous | None | Variant |
| Termination of supported period |  |  |  |
|  | Feels able to go back to treatment if needed | Typical | Typical |
|  | Happy with how support was terminated | General | None |
|  | Had a conversation with supporter about finishing treatment | Variant | Variant |
|  | No longer a priority, just let it go | Variant | Variant |
|  | Support stopped unexpectedly, felt abandoned | None | Variant |
|  | Felt relieved that support stopped as it was a negative experience | None | Variant |

^a^ Reported negative reasons for change in motivation to continue engaging with treatment are: not being in a receptive frame of mind, contextual obstacles and iCBT not considered to be personally fitting.

^b^ Only thirteen participants (five felt ready to leave treatment early and eight had negative reasons for their change in motivation) reported on the reasons for their change in motivation

^c^ General’ results apply to all cases (i.e. 5/5 and 8/8), ‘typical’ results apply to at least half of the cases (i.e. 3-4/5 and 5-7/8) and ‘variant’ results apply to fewer than half, of the cases (i.e. 1-2/5 and 1-4/8)
